# Pelvic Congestion Syndrome in a Postmenopausal Female

**DOI:** 10.7759/cureus.17444

**Published:** 2021-08-25

**Authors:** Neha Potla, Savithri-Chandana Veluri, Thor S Stead, Jesse Dubey, Latha Ganti

**Affiliations:** 1 Emergency Medicine, Unionville High School, Kennett Square, USA; 2 Women's Health, Orlando Veterans Affairs Medical Center, Orlando, USA; 3 Medicine, Warren Alpert Medical School, Providence, USA; 4 Emergency Medicine, Lakeland Regional Health, Lakeland, USA; 5 Emergency Medicine, Envision Physician Services, Plantation, USA; 6 Emergency Medicine, University of Central Florida College of Medicine, Orlando, USA; 7 Emergency Medicine, Ocala Regional Medical Center, Ocala, USA; 8 Emergency Medicine, HCA Healthcare Graduate Medical Education Consortium Emergency Medicine Residency Program of Greater Orlando, Orlando, USA

**Keywords:** pelvic congestion syndrome, menopausal, pelvic pain, pregnancy, estrogen, treatment, ovarian function, varicose vein

## Abstract

This study reports the case of a 53-year-old postmenopausal woman and explores her unique experience with pelvic congestion syndrome (PCS). PCS is a relatively newly recognized entity and is still a diagnosis of exclusion. We analyze the presenting symptoms and imaging findings on CT and ultrasonography. We also examine the potential causes of this ambiguous prognosis. This case is unusual in that PCS usually presents in premenopausal rather than postmenopausal women.

## Introduction

The scope of current knowledge on pelvic congestion syndrome (PCS) is sparse as it is a relatively newly recognized entity and a diagnosis of exclusion. PCS is a chronic condition that commonly affects females of childbearing age, especially women who have given birth to multiple children. It results in aching or sharp bursts of pelvic pain [1]. Although the syndrome is relatively new, the prevalence of the disease in women is nearly 30% as PCS is frequently misdiagnosed [2].

The cause of the congestion has yet to be elucidated; however, the etiology appears to be multifactorial. Currently, three speculations exist on its etiology: 1) valvular insufficiency of ovarian vein or internal iliac veins leading to stasis of blood, 2) venous outflow obstruction due to extrinsic compression of pelvic veins, and 3) vasodilatory effect of estrogen on pelvic vasculature. This case presentation challenges the third speculation. The condition is rarely seen in postmenopausal women because estrogen levels are lower in this age group. The main symptoms of PCS are abdominal pain on the left side more than the right that lasts for at least six months. Pain is typically exacerbated by factors that increase intra-abdominal pressure, such as prolonged standing, and is relieved by lying down. Pain worsens during menstrual periods, before and after coitus, and during pregnancy. PCS is associated with irritable bowel symptoms and stress incontinence. Left-sided pain is more common, perhaps due to a higher incidence of congenital absence of valves of the left ovarian vein. The left ovarian vein drains into the renal vein in contrast with the right ovarian vein that drains directly into the inferior vena cava. The pain is typically dull and exacerbated by general movement. There is often accompanying increased urinary frequency, abnormal menstruation, irritable bowel symptoms, and enlarged veins around the vulva area and pelvic floor [3]. The treatment options for PCS are embolization, gonadotropin-releasing hormone drugs to block ovarian function, progestin hormone drugs to relieve pain, and hysterectomy. Pelvic floor physical therapy is an adjunctive treatment that may help with coexisting pelvic floor dysfunction and abnormal bowel and bladder function. Behavioral therapy for associated conditions such as depression, anxiety, and sexual dysfunction can also be considered. PCS is a diagnosis of exclusion [4].

## Case presentation

A 53-year-old postmenopausal female presented with severe abdominal pain and bloating in the suprapubic area. The pain had started three days prior and was worse on the left side. Her surgical history was significant only for a cholecystectomy. Her only daily prescription was duloxetine 30 mg daily for the past year for anxiety related to coronavirus disease 2019 (COVID-19). She reported that the pain worsened when engaged in daily activities such as walking, a key symptom of PCS. She denied fevers, chills, chest pains, shortness of breath, nausea, vomiting, diarrhea, or constipation. Her vital signs included a blood pressure of 108/67 mmHg, temperature of 98.7 °F, pulse rate of 98 beats per minute, respiratory rate of 16 breaths per minute, and oxygen saturation of 99% on room air. Her physical exam was only remarkable for left-sided abdominal pain. Her laboratory analysis was unremarkable except for slight traces of blood and ketones in the urine (Table 1).

**Table 1:** Laboratory analysis of the patient BUN: blood urea nitrogen; GFR: glomerular filtration rate; AST: aspartate aminotransferase; ALT: alanine aminotransferase; ALP: alkaline phosphatase; WBC: white blood cell; RBC: red blood cell; Hgb: hemoglobin; Hct: hematocrit; MCV: mean corpuscular volume; MCH: mean corpuscular hemoglobin; MCHC: mean corpuscular hemoglobin concentration; RDW: red cell distribution width; MPV: mean platelet volume; COVID-19: coronavirus disease 2019; NAA: nucleic acid amplification

Test	Results	Reference range
Sodium (mmol/L)	141	135–145
Potassium (mmol/L)	3.5	3.5–5.3
Chloride	107	99–111
Carbon dioxide (mmol/L)	27	21–32
Anion gap	10.5	
BUN (mg/dL)	10	7–22
Creatinine (mg/dL)	0.6	0.6–1.3
Estimated GFR (MDRD)	>60	>60
Glucose (mg/dL)	89	70–110
Calcium (mg/dL)	8.4	8.4–10.2
Ionized calcium (mg/dL)	3.6	
Total bilirubin (mg/dL)	0.5	0.0–1.0
AST (Units/L)	12	7–37
ALT (Units/L)	19	12–78
Total ALP (Units/L)	56	50–136
Total protein (g/dL)	7.3	6.4–8.2
Albumin (g/dL)	3.5	3.4–5.0
Lipase (Units/L)	78	65–230
Hematology		
WBC (x 10^3^/uL)	8.9	4.0–10.5
RBC (x 10^6^/uL)	4.01	3.93–5.22
Hgb (g/dL)	11.7	11.2–15.7
Hct (%)	37.6	34.1–44.9
MCV (fL)	93.8	79.4–94.8
MCH (pg)	29.2	25.6–32.2
MCHC (g/dL)	31.1	32.2–35.5
RDW (%)	13.2	11.7–14.4
Platelet count (x 10^3^/uL)	233	150–400
MPV (fL)	9.5	9.4–12.4
Absolute basos (auto) (x 10^3^/uL)	0.03	0.01–0.08
Nucleated RBC (%)	0	0.0–0.2
Immature granulocytes (x 10^3^/uL)	0.02	0.00–0.03
Neutrophils (x 10^3^/uL)	7.01 H	1.56–6.13
Lymphocytes (x 10^3^/uL)	1.24	1.18–3.74
Monocytes (x 10^3^/uL)	0.48	0.24–0.6300
Eosinophils (x 10^3^/uL)	0.13	0.04–0.36
Nucleated RBCs (x 10^3^/uL)	0	0.00–0.18
Serology		
COVID-19 (NAA)	Negative	Negative
Urines		
Urine color	Yellow	Yellow
Urine appearance	Clear	Clear
Urine pH	5	5.0–8.0
Urine specific gravity	≥1.030	1.005–1.030
Urine protein (mg/dL)	Negative	Negative
Urine glucose (stick) (mg/dL)	Negative	Negative
Urine ketones (mg/dL)	1+ A	Negative
Urine blood	Track-intact A	Negative
Urine nitrite	Negative	Negative
Urine bilirubin	Negative	Negative
Urine urobilinogen (EU/dL)	0.2	0.2–1.0
Urine leukocyte esterase	Negative	Negative

**Figure 1:**
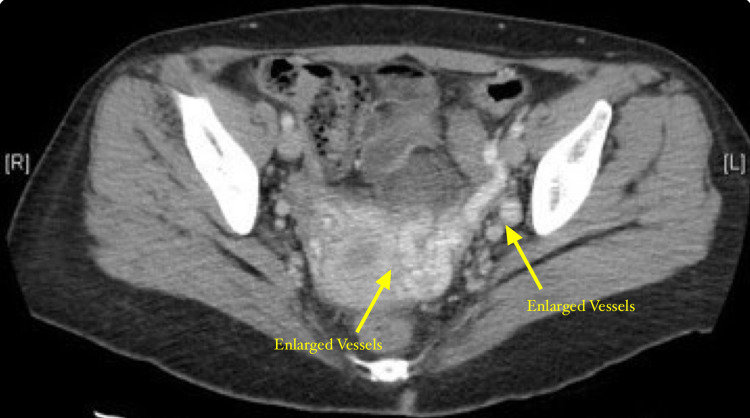
CT of the postmenopausal pelvis showing dilated vein structures surrounding the uterus and ovaries on the left CT: computed tomography

CT of the abdomen and pelvis revealed para-adnexal varicosities on the left greater than right (Figure 1).

**Figure 2:**
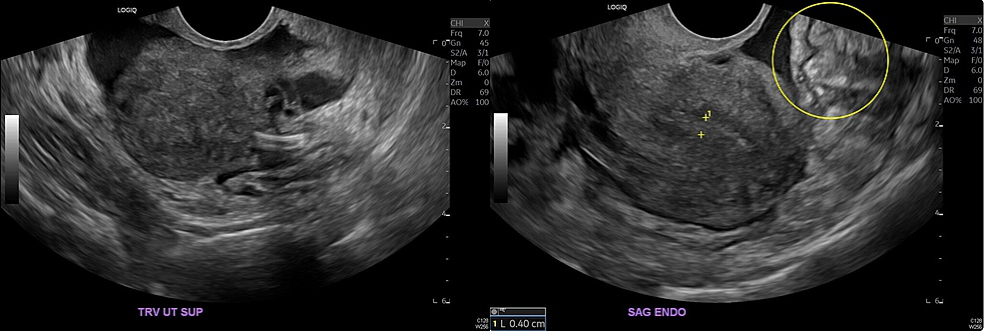
The Doppler ultrasound depicts the varicose veins prominent on the left and significant Doppler flow in the vessels (circle)

Pelvic ultrasonography revealed an unremarkable uterus with a normal 0.4-mm endometrial stripe. An implantable birth control device was visualized in the left uterine cornua. Both ovaries were noted to be of normal size (Figure 2).

**Figure 3:**
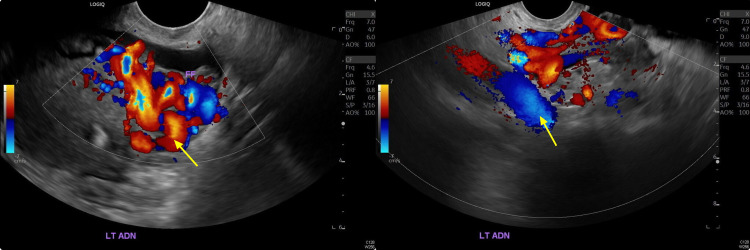
Color Doppler ultrasound images demonstrating extensive venous blood from the laminar with heavy flow towards and out of the uterus

There were multiple dilated veins in the adnexa with the reversed venous flow on color Doppler (Figure 3).

The patient was given intravenous ketorolac for analgesia, and she experienced relief. She was tentatively diagnosed with PCS based on the exquisite pain dominant on the left, increased pain when performing daily activities, and the findings of enlarged pelvic veins on imaging. She was discharged home with a follow-up arrangement in a clinic that specializes in treating PCS.

## Discussion

This particular case of PCS is unique as instances of postmenopausal females reporting pelvic congestion syndrome are sporadic at best. Women in menopause have less estrogen because the ovaries decrease their rate of production. Estrogen dilates the veins. During menopause, when estrogen levels are low, the pelvis’s blood supply decreases [5]. This is why vein fluid build-up is unlikely; however, for postmenopausal females, it is still possible to acquire PCS if the veins have been severely dilated.

The abnormality of our patient's case centered on her particular symptoms. She reported normal bowel movements, no dysuria, and no bulging veins around the thighs or vulva. Approximately 65-79% of women with PCS report irritable bowel syndrome (IBS) [6], but our patient did not. Similarly, patients with PCS often report urological symptoms like dysuria or painful urination, hematuria, and polyuria [1], and again our patient did not. She experienced no additional pain at the pelvic girdle or sacrum.

**Figure 4:**
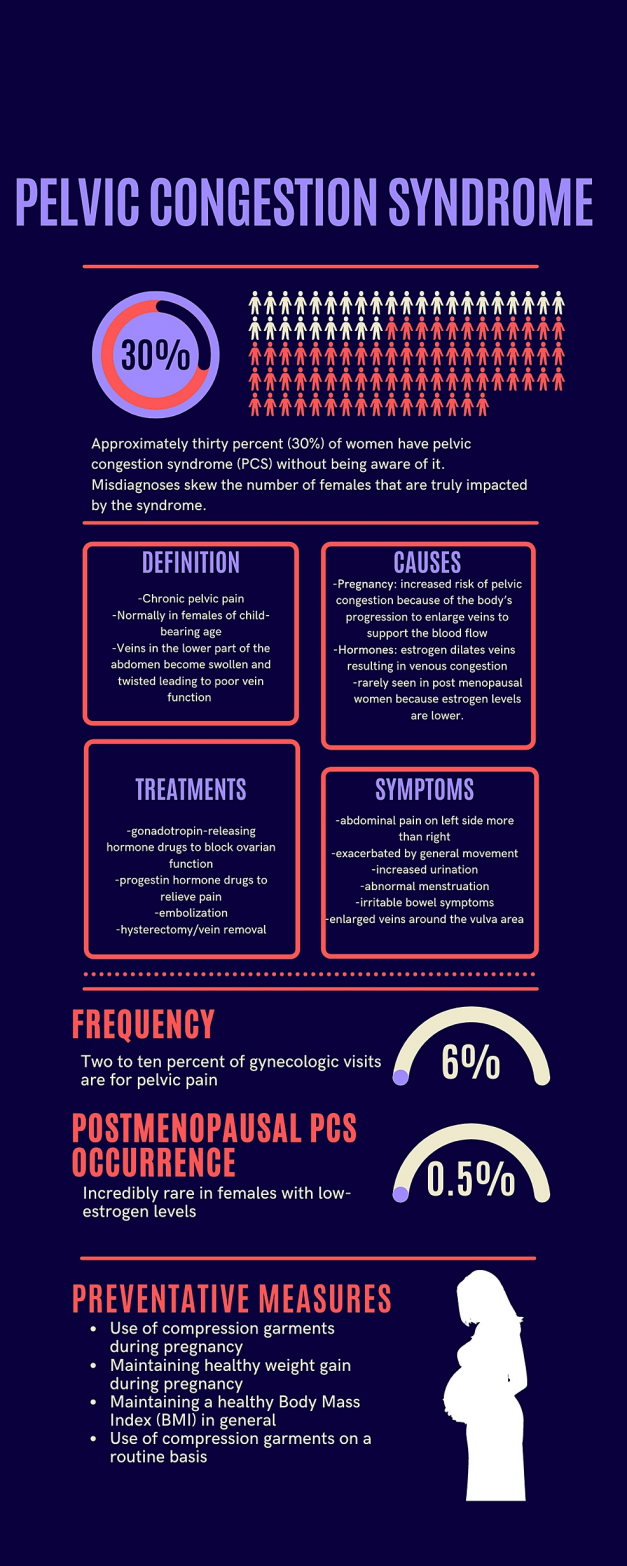
Infographic summarizing the main features of pelvic congestion syndrome

There are several options to treat PCS. The first option involves hormonal medication that can significantly reduce the blood flow to the veins and eventually minimize the prevalence of the varicose veins. The medication is either gonadotropin-releasing hormone, which blocks the function of the ovaries to relieve pain, or synthetic progestin hormone, which activates progesterone receptors to relieve pain. This option is the least invasive; however, if medication proves to be ineffective, there are minimally invasive procedures to alleviate the pain. The second option is nonsurgical embolization or sclerotherapy, which intentionally shuts off damaged veins and prevents further build-up. For the embolization, a catheter is inserted into a vein in the upper arm, shoulder, or thigh depending on the area of the problem and follows the vein to the source of the pain through radiographic guidance. Finally, the third procedural option is to surgically remove the damaged vein(s) or to have a hysterectomy [7]. Generally, the least invasive methods are attempted first (Figure 4).

There are two case reports of postmenopausal women experiencing PCS in the literature so far. Neither of those cases is similar to our patient's experience. The first one was in a 55-year-old woman with congenital varicosities [8]. The second one was in a 69-year-old postmenopausal patient with varicose veins, but she was asymptomatic. The lack of symptoms is very uncharacteristic of PCS [9]. Furthermore, other syndromes like May-Thurner appear to have similarities with PCS, which seem to be more applicable to patients experiencing menopause.

One interesting aspect to note in our patient’s case is the implantable birth control device noted on ultrasound. This is a permanently implanted metal birth control coil that was recalled in September of 2019 [10]. Another patient had presented with a dull lower abdominal pain after the insertion of this device. The blood urine in postmenopausal women needs to be further worked up. This issue led to the speculation of a complication arising from a urinary tract infection (UTI). This patient also explained that over time the pain had become constant and exacerbated during intercourse. The pelvic pain continued to worsen until she requested to have the device removed through a laparoscopic bilateral total salpingectomy. After removal, the patient conclusively reported that the chronic pelvic pain resolved itself [11]. It is possible that her pain was related to this device as well. It is likely that this device may have caused external compression of ovarian veins. It is also possible that the patient may have genetic factors like absent valves of ovarian veins that predisposed her to PCS. Patients with implantable birth control devices like the one our patient had are also known to have reported increased incidence of abnormal uterine bleeding as a common side effect, implying that in spite of menopause, the device could have caused a state of ‘hormonal dysfunction’ with relatively higher estrogen than progesterone, thereby increasing the risk of PCS. Indeed, many women have previously reported pelvic pain after being implanted with this device and litigated [12].

One other plausible diagnosis is May-Thurner syndrome (MTS), which occurs when the left iliac vein is compressed by its right iliac counterpart. This can increase the risk of deep vein thrombosis (DVT) or blood clot that causes pain on a slightly left trajectory. MTS is also less common in older females [13]. MTS can often be mistaken for PCS [14]. Both syndromes share areas of pain in the pelvic region.

## Conclusions

This case highlights the importance of obtaining a thorough history from the patient, and keeping a broad differential, even when symptoms are atypical or present in an atypical patient. PCS is a condition that can lead to significant morbidity due to the chronic, often disabling nature of the pain it causes. It is most commonly seen in premenopausal women. PCS is rarely reported in postmenopausal women, but it does occur, as discussed in this case report.
